# High Source Strength of Most Blister Packs Delivered for 125I Seed Brachytherapy: A Case Report

**DOI:** 10.7759/cureus.92023

**Published:** 2025-09-10

**Authors:** Masato Takanashi, Isao Kuroda, Shinji Sugahara, Masumi Kawaguchi, Masataka Hoshina, Masaya Noguchi, Koichi Masuda

**Affiliations:** 1 Department of Radiology and Radiation Oncology, Tokyo Medical University Ibaraki Medical Center, Inashiki-gun, JPN; 2 Department of Urology, Tokyo Medical University Ibaraki Medical Center, Inashiki-gun, JPN

**Keywords:** 125i seed brachytherapy, batch assay, low-dose-rate brachytherapy, prostate cancer, single-seed assay, well-type ionization chamber

## Abstract

Since the beginning of 125I seed brachytherapy, our facility has verified the source strength of all blister packs delivered. We set a reference range based on measurements taken with a well-type ionization chamber. There are scattered reports on verification for the purpose of detecting dead seeds, but to our knowledge, there are no reports on the application of source strength verification results in actual clinical practice for deviations of a few percent below the intervention value. In this case, five of the six TheraAgX100 (Theragenics Corporation, Buford, GA, USA) blister packs delivered had a source strength that was 3-5% stronger than the nominal value. We report on this case, in which we consulted with the physician regarding the placement of the source, taking into consideration the measured source strength, which led to a safer treatment. We used VariSeed 9.0 (Varian Medical Systems Co., Ltd., Palo Alto, CA, USA), a treatment planning system (TPS), with a nominal value of 0.274 mCi and a nominal value of 0.288 mCi, which is 5% stronger than the nominal value for safety reasons, and verified the difference in prescribed doses for planning target volume (PTV) and organ at risk (OAR). We placed 0.288 mCi in areas where the distance from the urethra and rectum, which are susceptible to adverse events, could be ensured. When comparing the nominal value and measured value of the prescribed dose, the measured value of PTV D_90_ increased by 2.73%. In addition, as indicators of the dose to the urethra, UD_10_ and UD_30_ increased by 3.04% and 2.64%, respectively, in the measured values. Next, as an indicator of the dose to the rectum, RD_0.1 cc_ increased by 3.43 Gy in the measured values. In radiation therapy, it is essential to accurately determine the prescribed dose. Evaluating the deviation in source strength in relation to the prescribed dose is not only useful for determining the effectiveness of treatment, but also enables the source to be left in place while minimizing the occurrence of adverse events. It is an important responsibility of medical physicists to provide the best possible treatment to patients by thoroughly conducting the verification that has been advocated and collaborating with physicians.

## Introduction

Prostate cancer is one of the most common malignant tumors in males, and its incidence is increasing worldwide [1]. Furthermore, both incidence and mortality rates are expected to double in the future [2].

In Japan, 125I seed brachytherapy was introduced in 2003 and has been used to treat many patients. Yorozu reports that 125I seed brachytherapy has demonstrated superior efficacy compared to other treatments such as radical prostatectomy and external beam radiation therapy in prospective and randomized trials [3]. Another advantage is that there have been few reports of adverse events [4,5].

125I seed brachytherapy is a treatment in which multiple small capsules containing the radioactive substance 125I are implanted into the prostate. Naturally, if the source strength of 125I differs from the nominal value presented by the manufacturer, it may affect the treatment outcome. According to guidelines in Europe and the United States, it is the responsibility of medical physicists to verify the source strength before treating patients [6-11]. Additionally, the Task Group (TG) report from the American Association of Physicists in Medicine (AAPM) recommends that users verify the source strength in addition to the single-seed assay conducted by the manufacturer [7,8].

According to a report by Kojima et al., there have been three cases in Japan since 2017 in which seeds with incorrect source strength were delivered to medical facilities [12]. Specifically, seeds with source strengths different from those ordered by medical facilities and dead seeds were delivered. This led to a loss of trust in the source strengths published by manufacturers. In a survey conducted in response to these incidents, only 16% of facilities had verified source strength, and only 52% had verified the number of sources. Reasons cited for the low number of facilities conducting verification included insufficient equipment for verification, lack of knowledge regarding verification procedures, shortage of staff to conduct verification, and the fact that many sources circulating in Japan cannot be re-sterilized.

Due to the factors mentioned above, it is difficult to perform a single-seed assay in Japan [12,13]. Therefore, batch assay, which enables the measurement of multiple sources in a sterile state, has been proposed as an alternative to single-seed assay [14-18]. Previous papers have reported methods using a well-type ionization chamber [14-16], an ionization chamber [17], and imaging plates [18]. The advantages of batch assay include high time efficiency and reduced exposure to the person doing the measurements. On the other hand, there's the downside that you cannot accurately evaluate the source strength of each individual source. However, dead seeds and sources with different examination dates, as mentioned above, can be detected, so it is considered a practical method in clinical practices.

## Case presentation

**Figure 1:**
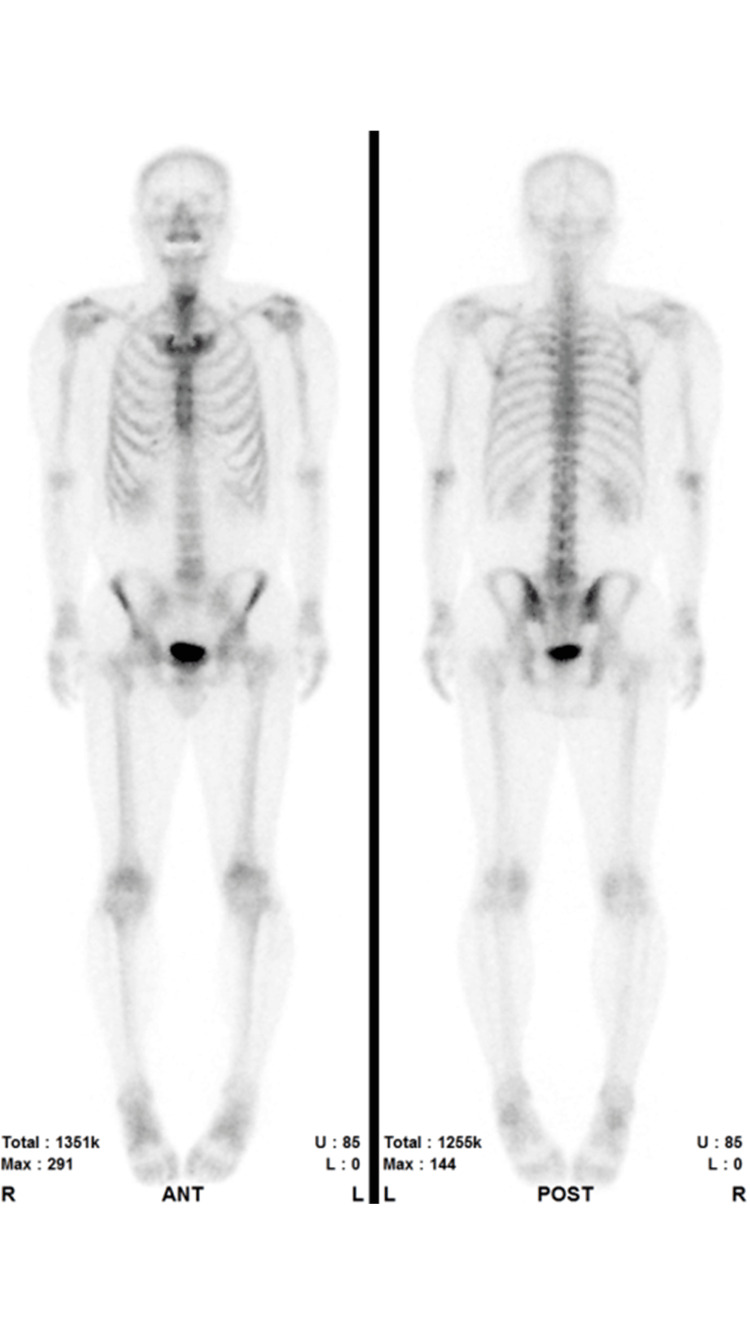
99mTC-MDP bone scintigraphy for preoperative metastasis screening. No accumulation suggestive of bone metastasis was observed on bone scintigraphy. 99mTC-MDP: technetium-99m-methylene diphosphonate.

The patient was a 50-year-old man with an initial prostate-specific antigen (iPSA) of 5.17. A tissue biopsy revealed adenocarcinoma with a Gleason score of 3+3 = 6. The patient was low risk (T1cN0M0) and opted for prostate-specific antigen (PSA) monitoring therapy. Subsequently, an increase in PSA was observed during follow-up. Imaging studies, including MRI, did not reveal any significant lesions. No suspicious bone metastases were detected on 99mTC-MDP (technetium-99m-methylene diphosphonate) bone scintigraphy (Figure 1). Based on the above findings, 125I seed brachytherapy was performed.

Summary of the case

As mentioned above, the AAPM advocates the importance of source strength verification at the facility where it is used. Since the start of 125I seed brachytherapy, our facility has been verifying the source strength of all blister packs delivered. The method used is to set a tolerance range based on measurements taken with a well-type ionization chamber. There are scattered reports on verification for the purpose of detecting dead seeds, but to our knowledge, there are no reports on the use of source strength verification results in actual clinical practice for deviations of ±5% or under, which is under the intervention value. Of the six sets of TheraAgX100 (Theragenics Corporation, Buford, GA, USA) blister packs delivered this time, five sets had a source strength that was 3-5% stronger than the nominal value. The guidelines recommend that for products with a deviation of more than 5% from the nominal value in batch measurements, the results of the measurements should be discussed with the physician. We report on a case in which, after considering the measured source strength and discussing the placement of the source with the physician, we were able to provide a highly safe treatment.

**Figure 2:**
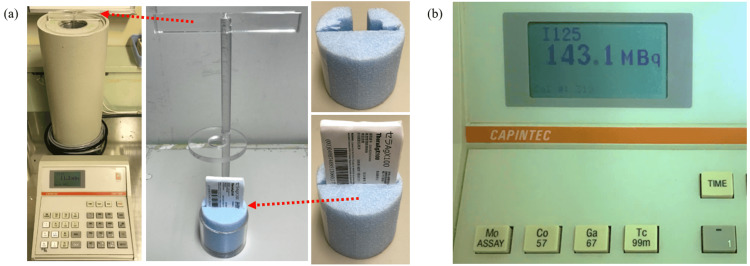
Method for measuring radiation source strength using a well-type ionization chamber (batch assay at our facility). At our facility, we verify source strength using a batch assay with a well-type ionization chamber. The well-type ionization chamber we use is the CRC-15R (Capintec Corp.). As a measurement procedure, we fix the blister pack to a homemade styrofoam pedestal, as shown in image (a). Next, it is attached to an acrylic hanging rod and placed in the well-type ionization chamber. The measured values are then read, as shown in image (b).

At our facility, we verify source strength using a batch assay with a well-type ionization chamber. The well-type ionization chamber we use is the CRC-15R (Capintec Corp., Florham Park, NJ, USA). As a measurement procedure, we fix the blister pack to a homemade styrofoam pedestal, as shown in Figure 2A. Next, it is attached to an acrylic hanging rod and placed in the well-type ionization chamber. The measured values are then read, as shown in Figure 2B.

The half-life of 125I is 59.4 days. In busy clinical practice, it is not always possible to perform measurements on the specified date and time. Therefore, when the measurement date differed from the examination date, decay correction was applied to the measured values. In Japan, it is mandatory by law to calibrate measuring instruments regularly. The well-type ionization chamber used in this case was calibrated regularly, and the measured values are reliable.

**Figure 3:**
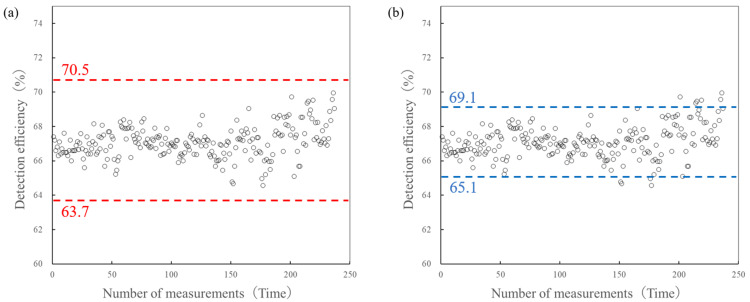
Set tolerance and intervention values based on the cumulative detection rate accumulated at the facility, and detect blister packs that exceed each value (example of 20 blister packs). The figure shows a scatter plot of the cumulative detection rates for 237 samples from 20 blister packs. The dotted line in image (a) indicates a line that deviates by 5% from the average detection rate. In our facility, this corresponds to 63.7-70.5%, and it can be seen that no samples deviate from this range. Therefore, it can be concluded that all samples are below the intervention value. Next, the dotted line in image (b) shows the line that deviates 3% from the average detection rate. In the case of our facility, this corresponds to 65.1-69.1%, and it can be seen that some samples exceed the tolerance value. As such, it can be seen that there is a certain number of blister packs that exceed the tolerance value.

The tolerance and intervention values for batch assay must be calculated from the cumulative detection rates at the facility where they are used. Figure 3 shows a scatter plot of the cumulative detection rates for 237 samples from 20 blister packs. The dotted line in Figure 3A indicates a line that deviates by 5% from the average detection rate. In our facility, this corresponds to 63.7-70.5%, and it can be seen that no samples deviate from this range. Therefore, it can be concluded that all samples are below the intervention value. Next, the dotted line in Figure 3B shows the line that deviates 3% from the average detection rate. In the case of our facility, this corresponds to 65.1-69.1%, and it can be seen that some samples exceed the tolerance value. As such, it can be seen that there is a certain number of blister packs that exceed the tolerance value.

In this case, a total of six sets of blister packs were delivered. The breakdown was three sets of 20 blister packs, two sets of five blister packs, and one set of four blister packs. Table 1 shows the measured values and average values for each blister pack. Of these, a total of five sets, excluding one set of 20 blister packs, had a source strength that was 3-5% stronger than the nominal value.

**Table 1:** Measured source strength of each blister pack.

	Measurement value (MBq)						Special note
Number of sources	1	2	3	4	5	Average	
20	144.4	144.4	144.5	144.4	144.4	144.4	+3-5%
20	145.2	145.2	145.2	145.1	145.2	145.2	+3-5%
20	143.3	143.3	143.3	143.2	143.2	143.3	Within tolerance
5	46.2	46.2	46.3	46.2	46.2	46.2	+3-5%
5	46.0	45.9	46.0	46.0	46.0	46.0	+3-5%
4	37.4	37.4	37.5	37.4	37.4	37.4	+3-5%

The manufacturer performs a single-seed assay for each source. Table 2 shows the results of a single-seed assay provided by the manufacturer. Specifically, in accordance with the TG report, the manufacturer verifies that each source strength is within ±6% and that the average of all source strengths is within ±5%. The source strength delivered in this case was 11.0 MBq. Therefore, the manufacturer has confirmed that each source strength is within the range of 10.34-11.66 MBq, and the average of all source strengths is within the range of 10.45-11.55 MBq. Additionally, the number of sources is also confirmed to be correct. As described above, products whose source strength validity has been verified by a single-seed assay in the United States are imported into Japan.

**Table 2:** Results of single-seed assay provided by the manufacturer.

Seed No.	MBq	Seed No.	MBq	Seed No.	MBq	Seed No.	MBq
1	11.3	21	11.6	41	11.1	61	11.5
2	11.5	22	11.4	42	11.3	62	11.5
3	11.2	23	11.6	43	11.5	63	11.5
4	11.3	24	11.5	44	11.4	64	11.4
5	11.3	25	11.4	45	11.4	65	11.5
6	11.6	26	11.6	46	11.3	66	11.3
7	11.6	27	11.5	47	11.6	67	11.5
8	11.6	28	11.2	48	11.4	68	11.6
9	11.5	29	11.6	49	11.5	69	11.4
10	11.3	30	11.5	50	11.3	70	11.5
11	11.6	31	11.1	51	11.6	71	11.6
12	11.5	32	11.5	52	11.5	72	11.5
13	11.0	33	11.3	53	11.6	73	11.5
14	11.6	34	11.4	54	11.6	74	11.6
15	11.5	35	11.5	55	11.6		
16	11.5	36	10.9	56	11.6		
17	11.4	37	11.6	57	11.6		
18	11.3	38	11.3	58	11.6		
19	11.1	39	11.2	59	11.4		
20	11.2	40	11.4	60	11.4	Average	11.4

**Figure 4:**
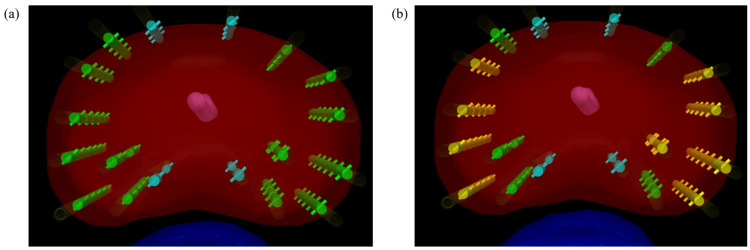
Source configuration of various source strengths in clinical practice. Discussing the placement of the radiation source to ensure safe treatment. Each structure in the figure is as follows: red represents the prostate, pink represents the urethra, and blue represents the rectum. The colors of the sources are as follows: green represents the nominal source strength, which is 11.0 MBq (0.274 mCi). Yellow indicates a source strength that is 3–5% higher than the nominal value. In this case, we considered safety and set it at 0.288 mCi, which is 5% higher. Blue indicates excess source strength. In this case, we set it at 0.232 mCi, which was corrected for decay from the examination date. Blue was also measured on a different day, and we confirmed that the source strength was within the tolerance range. Figure (a) shows the case where source strength is not taken into account, with only green and blue sources. Figure (b), on the other hand, takes into account a 3-5% difference in source strength, using three types of source strength, including yellow.

Each structure in Figure 4 is explained below. Red represents the prostate, pink represents the urethra, and blue represents the rectum. Next, the colors of the sources are explained. Green represents the nominal source strength, which is 11.0 MBq (0.274 mCi). Yellow indicates a source strength that is 3-5% higher than the nominal value. In this case, we considered safety and set it at 0.288 mCi, which is 5% higher. Blue indicates excess source strength. In this case, we set it at 0.232 mCi, which was corrected for decay from the examination date. Blue was also measured on a different day, and we confirmed that the source strength was within the tolerance range.

Figure 4A shows the case where source strength is not taken into account, with only green and blue sources. Figure 4B, on the other hand, takes into account a 3-5% difference in source strength, using three types of source strength, including yellow.

In this case, yellow sources with source strengths stronger than the nominal values were placed at the peripheral edge of the prostate, and green sources with source strengths close to the nominal values were placed inside the prostate close to the organ at risk (OAR). Four green sources were used in five lines so that they would not mix with the yellow sources. In addition, the blue excess sources, whose source strength was lower than the nominal value, were placed in the most inner part of the prostate, as they were especially close to the OAR. Based on the above, determining the appropriate placement position for each source strength prevented an increase in the dose to the OAR, such as the urethra and rectum, and led to a highly safe treatment that minimized the occurrence of adverse events.

## Discussion

**Figure 5:**
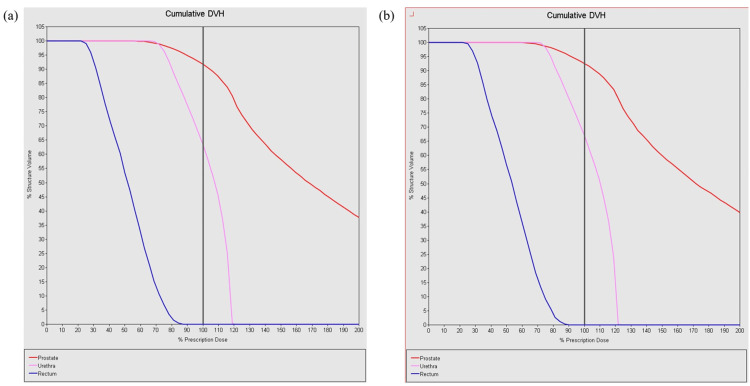
Difference in prescribed dose when taking into account a 3-5% difference in source strength (comparison of DVH curves). Figure (a) shows the dose volume histogram (DVH) curve without taking into account differences in source strength. Figure (b) shows the DVH curve taking into account differences in source strength of 3-5%. Each line represents the prostate (red), urethra (pink), and rectum (blue). Focusing on the organ at risk, it can be seen that the maximum dose to the urethra is below 120% in figure (a), but exceeds 120% in figure (b). Similarly, the maximum dose to the rectum is approximately 85% in figure (a), but approximately 90% in figure (b).

Figure 5A shows the dose volume histogram (DVH) curve without taking into account differences in source strength. Figure 5B shows the DVH curve taking into account differences in source strength of 3-5%. Each line represents the prostate (red), urethra (pink), and rectum (blue). Focusing on the OAR, it can be seen that the maximum dose to the urethra is below 120% in Figure 5A, but exceeds 120% in Figure 5B. Similarly, the maximum dose to the rectum is approximately 85% in Figure 5A, but approximately 90% in Figure 5B.

The guidelines provide several dose indexes for 125I seed brachytherapy. Table 3 shows each dose index. Planning target volume (PTV) D_90_, with the prostate as the PTV, is used as an index to determine the therapeutic effect, and 100-130% is recommended. Next, as indices for determining adverse events, there are UD_10_ and UD_30_ for the urethra, and values of under 150% and under 125% are recommended, respectively. In addition, there is RV_100_ for the rectum, and a value of under 1 mL is recommended. Furthermore, there is RD_0.1 cc_ for the rectum, which means the maximum dose for the rectum.

**Table 3:** Difference in prescribed dose when taking into account a 3-5% difference in source strength (comparison of dose indexes). Planning target volume (PTV) D_90_: 90% of the PTV volume receives the minimum radiation dose. UD_10_: The minimum radiation dose received is 10% of the urethral volume. UD_30_: The minimum radiation dose received is 30% of the urethral volume. RV_100_: Rectal volume irradiated with 100% or more of the prescribed dose. RD_0.1 cc_: The minimum radiation dose received by the rectum is 0.1 cc.

Dose index	Recommended value	Nominal value	Measurement value
PTV D_90_ (%)	100-130%	104.27	107.00
UD_10_ (%)	Under 150%	117.54	120.58
UD_30_ (%)	Under 125%	114.60	117.24
RV_100_ (mL)	Under 1 mL	0.00	0.00
RD_0.1 cc_ (Gy)	Under prescription dose	126.46	129.89

In this case, we compared the results obtained using only the nominal source strength with those obtained after taking into account a 3-5% difference in source strength. When the 3-5% difference in source strength was taken into account, PTV D_90_ increased by 2.73%. Furthermore, UD_10_ and UD_30_ increased by 3.04% and 2.64%, respectively. RV_100_ was 0.0 mL in both cases. RD_0.1 cc_ increased by 3.43 Gy.

**Figure 6:**
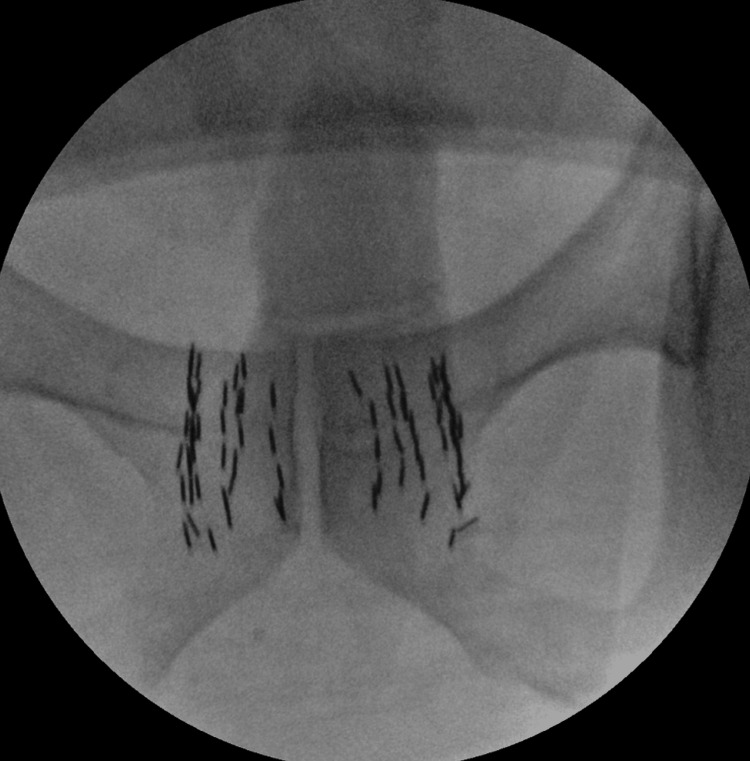
Confirmation of source distribution using postoperative X-ray fluoroscopy images.

Postoperative X-ray fluoroscopic images confirmed that each source was aligned neatly within the prostate (Figure 6).

In this case, the prescribed doses for the PTV and OAR changed when actual measured values were entered instead of nominal values. It has been reported that a 5% difference in prescribed dose can affect treatment outcomes. Therefore, it is clinically very important to obtain more accurate prescribed doses through source accuracy management.

At our facility, we perform source strength verification for all cases, with the primary objective being to detect sources with different calibration dates or dead seeds. However, even if the results of the batch assay fall below the intervention range, in cases like this one, where there are many blister packs with ±3-5% deviations, it is advisable to consider the source strength discrepancies.

## Conclusions

In radiation therapy, it is essential to accurately determine the prescribed dose. In this case, not only did we verify the source strength of all delivered blister packs, but since many of them exceeded the acceptable values, we evaluated the differences by inputting them into the treatment planning system. Evaluating the deviation in source strength in relation to the prescribed dose is not only useful for determining the effectiveness of treatment, but also enables the placement of the source in a way that minimizes the occurrence of adverse events. In this case, we reported on a case in which we were able to provide the best treatment for the patient by thoroughly conducting the verification, which is considered to be important, and discussing the appropriate placement position with doctors, medical physicists, and other professionals. It is an important responsibility of facilities that perform 125I seed brachytherapy to conduct source strength verification as recommended in the AAPM TG report. We will continue to verify source strength to provide safe treatment.
